# Effects of exercise snacks on physical and mental health among sedentary students: a randomized controlled trial study protocol

**DOI:** 10.3389/fpsyt.2026.1863839

**Published:** 2026-08-12

**Authors:** Zhenbin Bao, Zezhi Li, Tong Han, Lin Mi, Guoping Qian, Baoguo Li, Zbigniew Ossowski, Sujie Mao, Yintao Niu, Tao Lei, Jing Li, Jike Bai

**Affiliations:** 1 Department of Physical Culture, Gdansk University of Physical Education and Sport, Gdansk, Poland; 2 The Affiliated Brain Hospital of Guangzhou Medical University, Guangzhou, China; 3 Guangdong Engineering Technology Research Center for Translational Medicine of Mental Disorder, Guangzhou, China; 4 College of Physical Education, Shihezi University, Shihezi, China; 5 Graduate School, Harbin Sport University, Harbin, China; 6 Dongguan Mental Health Center of Southern Medical University (Dongguan Seventh People’s Hospital), Dongguan, China

**Keywords:** exercise prescription, exercise snacks, health promotion, sedentary behavior, university students

## Abstract

Sedentary behavior is highly prevalent among university students and is associated with adverse cardiometabolic, inflammatory, and psychological outcomes. Exercise snacks (ES), generally conceptualized as brief, structured bouts of physical activity distributed across the day, have emerged as a time-efficient strategy for improving health, but evidence in sedentary university students remains limited. This study is the initial phase of a multiphase randomised controlled trial program and aims to evaluate the effects of a structured, campus-based stair climbing ES intervention on the physical and mental health of sedentary university students. In this two-arm, parallel-group, superiority trial with blinded outcome assessment and blinded data analysis, 60 sedentary university students will be randomly allocated to an ES group or a usual-lifestyle control group. The intervention consists of a 12-week structured stair-climbing ES programme, including an initial adaptation phase and an intensified progression phase. The primary outcome is maximal oxygen uptake (VO_2_max). Secondary outcomes comprise mental health and selected physical health measures, whereas exploratory outcomes comprise metabolic, inflammatory, and adipokine biomarkers. Intention-to-treat analyses will use analysis of covariance (ANCOVA) or log-ANCOVA, with primary inference based on baseline-adjusted between-group differences in VO_2_max. This trial will test a pragmatic, campus-based intervention in sedentary university students and may provide methodological and empirical support for campus-based student health promotion and subsequent experiments within the registered program.

**Trial registration:**

https://www.chictr.org.cn/indexEN.html, identifier ChiCTR2500106549.

## Introduction

Physical inactivity (PI) and sedentary behavior (SB) are major modifiable behavioral risk factors contributing to the global burden of non-communicable diseases (NCDs) (1). The World Health Organization (WHO) identifies insufficient physical activity as a major contributor to global mortality, with an estimated 4–5 million preventable deaths annually (2, 3). Without effective interventions, PI-driven preventable NCD cases may rise to around 500 million by 2030 (4). Prolonged SB is strongly linked to adverse health events like heart disease and type 2 diabetes (5). Evidence indicates a dose-response relationship between SB and health risks: adults engaging in daily SB exceeding 6 hours experience a 26.7% elevated risk of 12 non-communicable diseases (e.g., heart disease, depression) relative to those with less than 2 hours (6). Adults with ≥ 8 hours of daily SB show an 112% higher risk of diabetes and a 147% higher risk of heart disease (7). These findings highlight the public health relevance of contemporary high-sedentary, low-activity lifestyles characterized by prolonged sedentary exposure and insufficient physical activity.

University students represent a relevant target population for campus-based lifestyle intervention research because the transition to university is accompanied by greater behavioral autonomy, prolonged academic sitting, screen-based learning, and irregular daily routines (8, 9). Available evidence suggests that university students spend an average of 10.69 h/day in sedentary behavior (10), while 44.4% fail to meet recommended physical activity levels (11). This evidence supports treating SB and physical activity as related but distinct intervention targets. Because movement behaviours may remain modifiable during the transition to independent campus life, this period provides a relevant window for early lifestyle intervention. In this population, sustained sedentary exposure combined with insufficient physical activity may contribute to unfavorable physiological and psychological risk profiles, including lower cardiorespiratory fitness (CRF) (12), dysregulated glycolipid metabolism (13, 14), increased chronic inflammation (15, 16), adipokine imbalance (17), and emotional disturbances (18, 19). These pathways are relevant to near-term outcomes such as excess adiposity, cognitive performance, and emotional regulation, and may contribute to longer-term NCD risk accumulation (20, 21). Taken together, these behavioral, health-related, and setting-specific characteristics support the evaluation of time-efficient, campus-based exercise strategies in university students.

High-intensity interval training (HIIT) can provide benefits similar to those of conventional moderate-intensity continuous training, including improved CRF and other health indicators (22–25). Although effective, many HIIT protocols require dedicated exercise time, structured sessions, and specific scheduling, which may limit their implementation in real-world campus routines (26, 27). Against this background, exercise snacks (ES) have emerged as a time-efficient physical activity strategy in which brief, structured bouts of activity are distributed across the day rather than completed as a single dedicated exercise session (28, 29). This distributed short-bout model is consistent with the broader framework of short bouts of accumulated exercise and provides a rationale for testing a structured stair-climbing ES intervention in sedentary university students (29, 30). In particular, stair-climbing ES may provide a practical campus-based modality because short bouts can be performed using existing built environments while still allowing a structured and reproducible exercise prescription (29, 31). An expanding body of evidence has supported the scientific rationale and potential effectiveness of ES and related short-bout accumulated exercise approaches (29, 31, 32).

Early randomized controlled trial (RCT) examined brief pre-meal exercise bouts and reported improved glycemic control in insulin-resistant adults (33, 34). Subsequent studies have tested ES or related short-bout accumulated exercise approaches, including stair climbing, cycling, resistance-based exercise, and Tai Chi, across healthy adults, older adults, and clinical populations (35–40). Existing reviews and meta-analyses suggest potential benefits for CRF and selected cardiometabolic outcomes, although effects appear to vary by population, exercise mode, dose, and outcome domain (41, 42). However, critical gaps remain in ES research targeting university students.

Specifically, current ES research shows a critical evidence gap in high-sedentary-risk student populations, with insufficient attention to psychological experiences and behavioral adherence (31). To address these gaps, this research program will systematically develop and evaluate a campus-based ES intervention. The primary aim of the present trial is to estimate the effect of the campus-based ES intervention on CRF. Secondary aims are to assess prespecified mental health indicators, blood pressure, body anthropometry, body composition, and vital capacity. Glycolipid metabolism, inflammatory markers, and adipokines will be assessed as exploratory mechanistic outcomes. Given its short duration and potential compatibility with campus routines, this initial phase is intended to evaluate a structured stair-climbing ES approach for university students with high sedentary exposure and low physical activity and to provide methodological and empirical support for subsequent campus-based student health-promotion studies.

## Methods and analysis

### Study design

**Figure 1:**
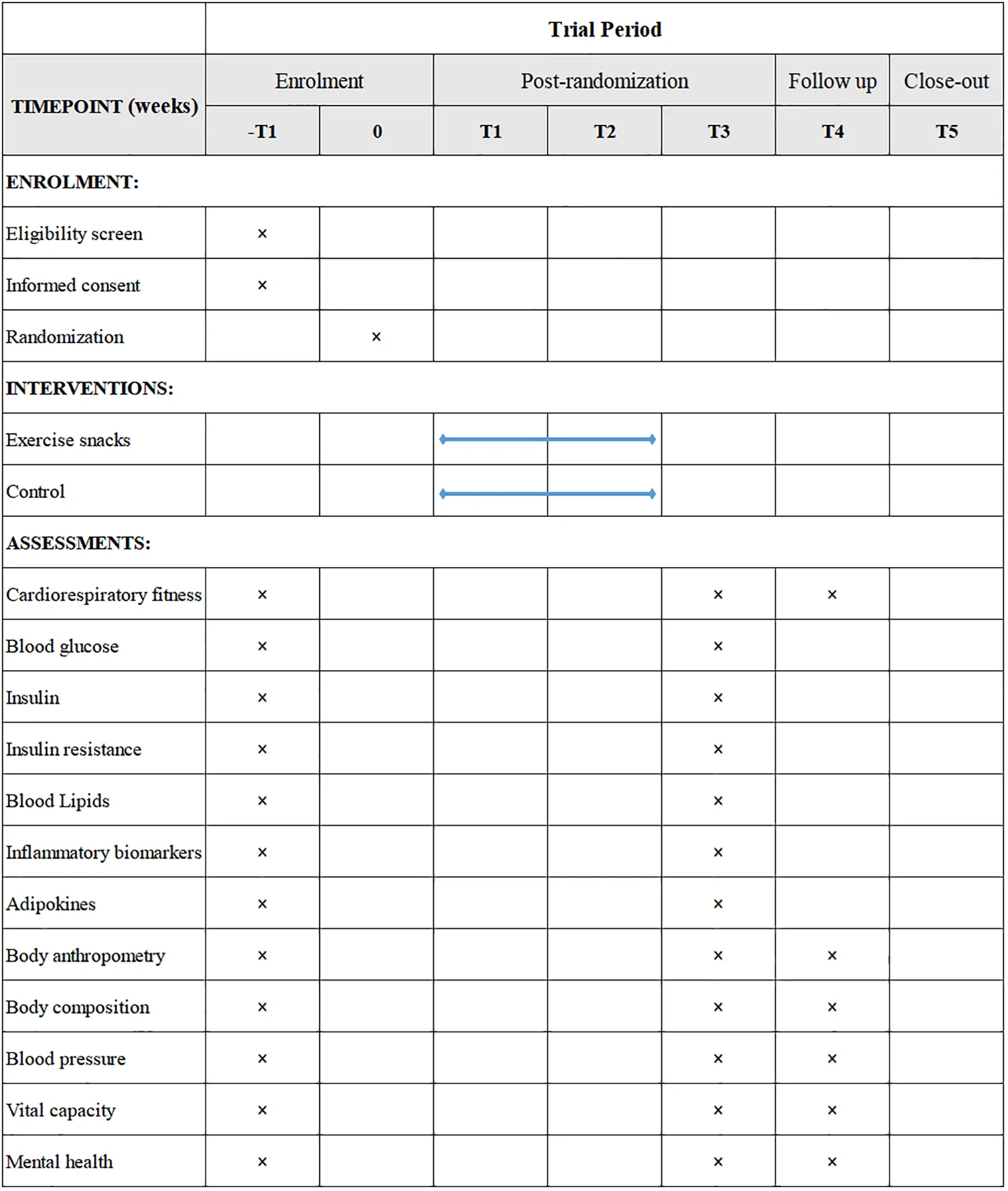
Trial schedule of enrolment, intervention, and outcome assessments. The timeline shows pre-randomization screening and baseline assessment (−T1), randomization (T0), supervised intervention delivery (T1–T3), post-intervention follow-up (T4), and trial close-out (T5). Crosses indicate prespecified assessments at the corresponding trial time points. T, trial time point.

**Figure 2:**
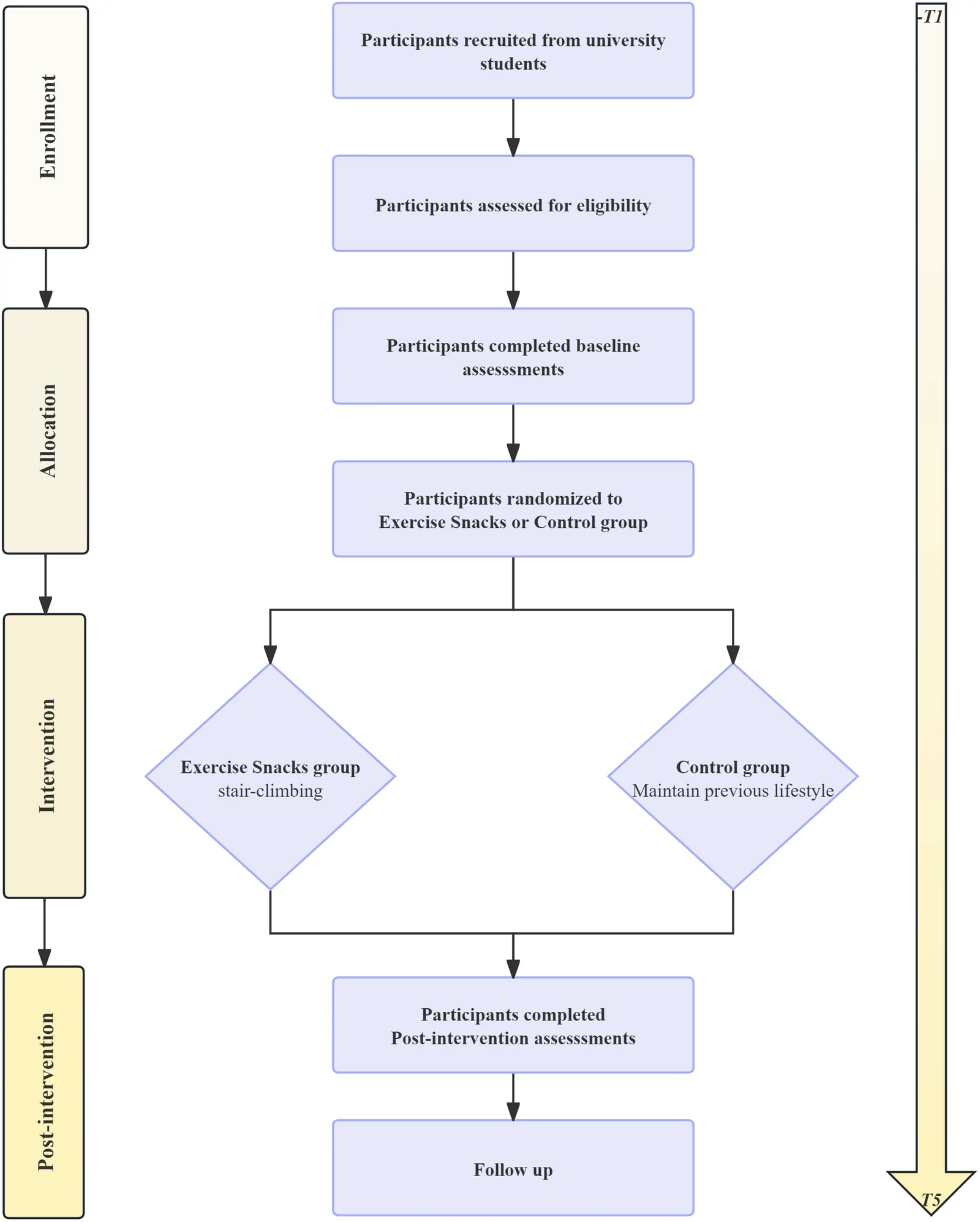
Participant flow diagram for the two-arm randomized controlled trial. The diagram summarizes participant recruitment, eligibility assessment, baseline assessment, random allocation to the exercise-snacks or control group, intervention delivery, post-intervention assessment, and follow-up. T, trial time point.

This study is the first experiment within a prospectively planned, multiphase randomized controlled trial program and is designed as a two-arm, parallel-group, superiority trial with 1:1 allocation, blinded outcome assessment, and blinded data analysis. The trial is registered under an overarching Chinese Clinical Trial Registry record (ChiCTR2500106549), which includes six prespecified experiments conducted under a single registration number and a shared prespecified outcome framework. Within this registered program, the recruitment dates reported in the registry refer to the recruitment period for the present experiment described in this protocol. The present experiment is designed to evaluate the effects of a 12-week ES intervention on physical and mental health outcomes in sedentary university students. Subsequent experiments within the registered program will be recruited separately in accordance with their respective protocols and prespecified eligibility procedures. Participants allocated to the ES group will undertake a 12-week progressive stair-climbing intervention. During the trial period, control participants will maintain their usual lifestyle and complete the same scheduled assessments. The protocol was developed in accordance with the SPIRIT 2025 statement (**Figure 1**; Additional file 1) (43), and the participant flow diagram is presented in **Figure 2**.

### Study setting

The Dongguan Seventh People’s Hospital initiated this study. The College of Physical Education of Shihezi University will carry out the study and serve as the implementing institution for the exercise intervention.

The study settings are as follows:

- Physiological and psychological assessments will be performed in the exercise performance, human morphology, and sports psychology laboratories of the School of Physical Education.
- Blood sample collection and subsequent specialized biochemical analyses will be conducted at an ISO 15189-certified laboratory.
- The exercise intervention will be implemented in designated campus stairwells located on the eastern and western sides of an academic building at Shihezi University. Both stairwells have a five-story structure with comparable step dimensions (step height: 15 ± 2 cm). These locations were selected to support campus accessibility, safe instructor supervision, comparable stair-climbing conditions, and reproducible delivery of the intervention protocol.

### Eligibility criteria

Inclusion criteria include the following:

- Full-time university students aged 18–32 years.
- Low self-reported physical activity, defined as ≤1.5 h/week of moderate-to-vigorous physical activity (MVPA), and high sedentary exposure, defined as ≥6 h/day of sedentary time, assessed using the International Physical Activity Questionnaire (IPAQ) (44).
- Completion of a health history questionnaire (45), Physical Activity Readiness Questionnaire (PAR-Q+) (46) and electrocardiogram screening to exclude potential cardiac rhythm abnormalities prior to enrollment.
- Provision of written informed consent and willingness to comply with the intervention and assessment procedures.

Exclusion criteria include the following:

- Self-reported MVPA exceeding the eligibility threshold, or participation in additional structured exercise training outside compulsory physical education classes.
- Current or recent musculoskeletal, cardiovascular, respiratory, metabolic, neurological, or psychiatric conditions that may contraindicate vigorous stair-climbing exercise or limit safe participation.
- Use of medications or treatments that may substantially affect cardiovascular, metabolic, inflammatory, or psychological outcomes.
- Majoring in sports science or exercise-training-related disciplines, to minimize confounding from discipline-specific curricular training, exercise literacy, habitual structured-activity exposure, and potential intervention contamination.
- Inability to comply with the intervention schedule, assessment procedures, or follow-up requirements.

### Who will take informed consent?

Potential participants will receive a face-to-face explanation of the study, and written informed consent will be obtained by trained members of the research team before enrolment and before any study-related procedures are undertaken.

### Additional consent provisions for collection and use of participant data and biological specimens

All collection of participant information, biological samples, and imaging data required for this study is covered in the formal informed consent form, and researchers will provide full disclosure during enrollment.

### Patient and public involvement

Patients and/or the public were not involved in the design, execution, reporting, or dissemination strategies for this study.

### Interventions

#### Explanation for the choice of comparators

A usual-lifestyle control group will be used as a non-exercise comparator to estimate the effects of the structured stair-climbing ES intervention. The same dietary-recording procedure will be applied to both groups. Self-reported Physical activity and sedentary time will be assessed using the IPAQ at baseline and post-intervention, with no continuous device-based monitoring of free-living physical activity or sedentary behavior planned during the 12-week trial. Trial-related contact with control participants will be limited to assessment scheduling, safety reporting, and completion of required study records.

#### Intervention description

**Figure 3:**
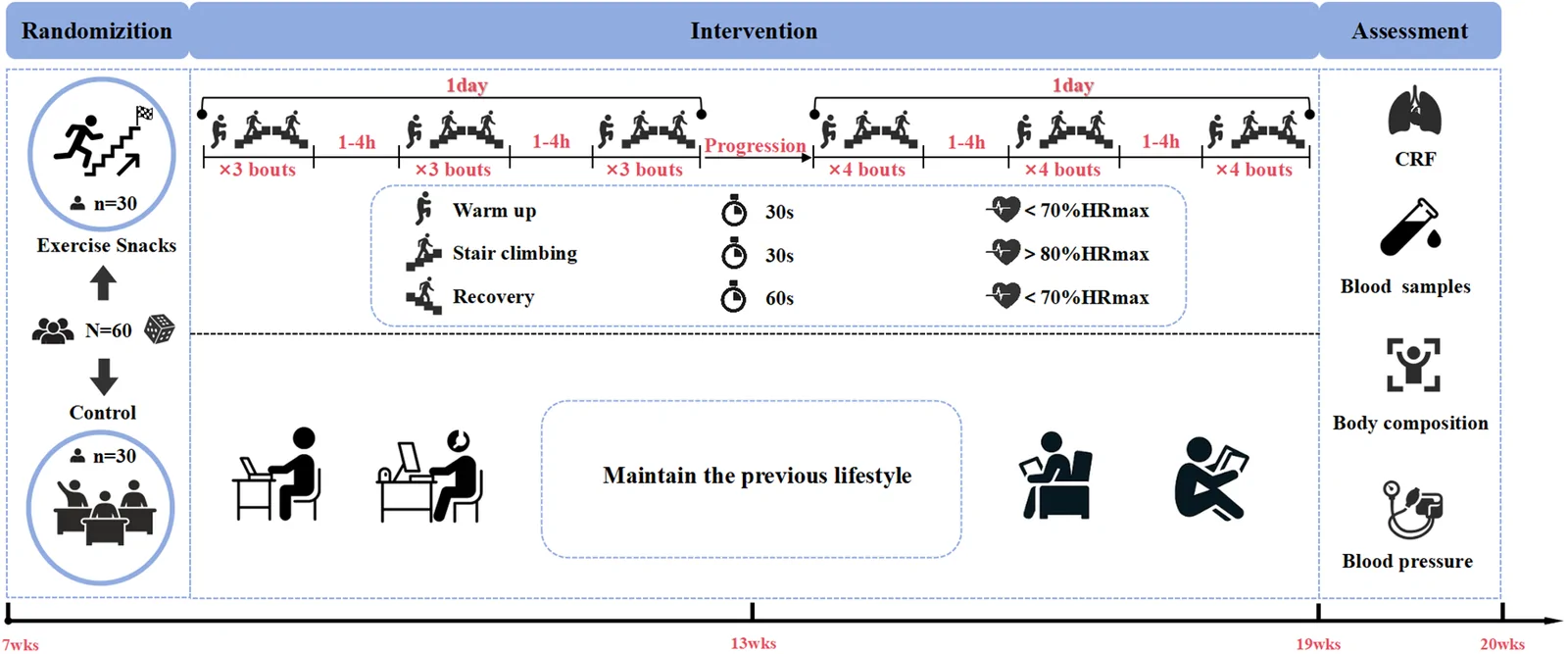
Overview of group allocation, intervention delivery, and outcome assessment. Participants are allocated to the exercise-snacks group or control group. The diagram summarizes the stair-climbing exercise-snack regimen, including warm-up, stair-climbing bouts, active recovery, progression from 3 to 4 bouts, between-session intervals, and common assessment procedures. CRF, cardiorespiratory fitness; HRmax, age-predicted maximal heart rate.

The ES group will receive a 12-week structured stair-climbing ES intervention (**Figure 3**; **Table 1**). The prescription was developed with reference to prior ES and related short-bout exercise studies summarized in **Table 2**, while the final FITT structure was aligned primarily with Yin et al. (41). Specifically, Yin et al. informed the adopted weekly frequency, within-day distribution, 30-s stair-climbing unit, between-session spacing, stair-climbing mode, and vigorous intensity-monitoring framework. The 12-week intervention duration was selected with reference to longer-duration ES evidence, including Zhou et al. (42). Overall, the adopted prescription was selected to balance feasibility, safety, and a sufficient vigorous stair-climbing stimulus in sedentary university students. In the present protocol, ES is operationalized as a distributed short-bout stair-climbing intervention within the broader framework of short bouts of accumulated exercise. The unit of within-day distribution is the short stair-climbing session: participants complete three sessions on each intervention day, separated by 1–4 h. Within each session, three to four 30-s vigorous stair-climbing sets separated by active recovery constitute the prescribed stair-climbing dose. Thus, the protocol preserves the distributed within-day structure of ES while transparently specifying the clustered set structure used to deliver a standardized stair-climbing stimulus.

**Table 1:** FITT-based prescription of the stair-climbing exercise-snack intervention.

Phase	Weeks 1–6	Weeks 7–12
Frequency	3 days/week; 3 sessions/day; 3 bouts × 30 s/session	3 days/week; 3 sessions/day; 4 bouts × 30 s/session
Intensity	80%–95% HRmax	80%–95% HRmax
Time	90 s/session; 270 s/day; 810 s/week	120 s/session; 360 s/day; 1080 s/week
Type	Stair climbing	Stair climbing
Volume	54 sessions; 162 bouts; 4860 s	54 sessions; 216 bouts; 6480 s
Progression	Initial adaptation phase with 3 bouts/session	Progression phase with 4 bouts/session after Week 6

FITT, frequency, intensity, time, and type; HRmax, maximal heart rate; s, seconds. Exercise time refers to accumulated stair-climbing sprint time and excludes active recovery. Exercise time and phase volume refer to accumulated stair-climbing sprint time and exclude active recovery. The control group maintained usual lifestyle and received no prescribed ES intervention during Weeks 1–12.

**Table 2:** Prior exercise-snack and related short-bout exercise studies considered during intervention development.

Study	Context and mode	Key protocol features	Duration/window	Role in present protocol
Francois et al. (34)	Individuals with insulin resistance; incline treadmill walking	Exercise snacking consisted of 6 × 1-min work bouts at approximately 90% HRmax, with 1-min slow-walking recovery between bouts, completed 30 min before meals	Acute crossover exercise-trial design	Provided contextual evidence for time-distributed planned exercise snacks; not used as the primary source for the final stair-climbing FITT structure
Allison et al. (35)	Previously untrained women; brief intense stair climbing	Brief intense stair-climbing protocols included repeated 20-s or 60-s stair-climbing efforts performed as vigorous or all-out exercise	6-week training protocols	Provided contextual evidence that brief intense stair-climbing protocols can improve cardiorespiratory fitness
Jenkins et al. (36)	Sedentary young adults; stair-climbing exercise snacks	Participants performed 3 bouts/day of vigorous stair climbing, separated by 1–4 h, 3 days/week	6 weeks	Supported the use of distributed stair-climbing bouts across the day, 1–4 h recovery intervals, and 3 days/week training frequency
Little et al. (37)	Healthy inactive young adults; cycle-ergometer sprint snacks	Sprint snacks involved 3 × 20-s all-out cycling bouts separated by 1–4 h of rest	18 training sessions over 6 weeks	Provided contextual evidence for distributing brief vigorous bouts across the day; modality was not adopted
Rafiei et al. (38)	Young healthy-weight men and adults with overweight/obesity; stair-climbing sedentary-break protocol	Stair-climbing snacks consisted of repeated 15–30-s stair-climbing bouts performed once per hour during a prolonged sitting condition	Acute randomized crossover studies	Provided contextual evidence for interrupting prolonged sitting with brief stair-climbing snacks; not used as a source for the long-term training prescription
Liang et al. (39)	Pre-frail older adults; home-based exercise and Tai-chi snacking	Progressive home-based strength exercise snacking and Tai-chi exercise snacking were delivered as short-bout exercise formats	12 weeks	Provided contextual evidence for longer-duration progressive exercise-snacking delivery; modality and population differed from the present stair-climbing protocol
Stork et al. (40)	Workplace adults; stair-climbing exercise snacks and stair-climbing HIIT	Stair-climbing exercise snacks involved brief isolated stair-climbing bouts performed across the day; the comparator stair-climbing HIIT condition used clustered bouts within a structured workout	Supervised exercise trials with workplace follow-up	Provided contextual evidence for practical delivery and receptivity of stair-climbing exercise snacks in real-world settings
Yin et al. (41)	Healthy inactive adults; stair-climbing exercise snacks	ES involved 30-s stair-climbing bouts distributed across the day, separated by at least 1 h, performed 3 days/week, with vigorous heart-rate responses	6 weeks	Primary source for the adopted weekly frequency, within-day distribution, 30-s stair-climbing unit, between-session spacing, stair-climbing mode, and vigorous intensity-monitoring framework
Zhou et al. (42)	Sedentary obese adults; stair-climbing exercise snacks	Participants performed stair-climbing exercise snacks 4 days/week for 12 weeks; each snack involved ascending four flights of stairs, 6 times/day, with intervals >1 h	12 weeks	Informed the 12-week duration and VO_2_max-based sample-size assumptions; the specific prescription of 4 days/week, 6 sessions/day, and 60 steps/session was not adopted

This table summarizes prior exercise-snack and related short-bout exercise studies considered during intervention development, including studies differing in design, duration, exercise mode, and population. The final column describes how each study informed the present protocol. FITT, frequency, intensity, time, and type; HRmax, maximum heart rate; HIIT, high-intensity interval training.

The intervention follows a two-phase dose-progression design to accommodate the gradual familiarization of sedentary university students with vigorous short-bout stair climbing.

- Basic adaptation phase (Weeks 1–6): Participants will complete three sessions per day on three days per week. Each session will comprise three 30-s vigorous stair-climbing sets, corresponding to 90 s of effective stair-climbing time per session. Sets will be performed as rapidly as safely possible, with a target intensity of 80%–95% of predicted maximum heart rate (HRmax), estimated as 208 − 0.7 × age (47). This phase is intended to familiarize participants with the stair-climbing protocol, support tolerance to vigorous short-bout exercise, and establish regular participation within campus routines.
- Intensive progression phase (Weeks 7–12): The target intensity, daily session frequency, and weekly frequency will remain unchanged. The number of 30-s stair-climbing sets will increase from three to four per session, corresponding to 120 s of effective stair-climbing time per session. This progression is intended to increase the total stair-climbing dose while preserving the same distributed within-day structure and recovery pattern (48).

The protocol is designed for implementation in a campus setting. Participants will complete the prescribed sessions within predefined daily time windows scheduled around their academic timetables to reduce scheduling conflicts and unnecessary travel burden. A 1–4 h interval will be maintained between sessions to allow recovery between distributed exercise exposures and to reduce cumulative fatigue. All sessions will be conducted in designated stairwells under instructor supervision and in accordance with the prespecified stair-climbing protocol. After each 30-s stair-climbing set, participants will complete 60 s of active recovery by slowly descending the stairs.

To ensure consistency in intervention delivery and intensity monitoring, the following procedures will be implemented (**Table 3**):

**Table 3:** Monitoring the training intensity in the exercise snacks group.

Intervention component	Prescribed duration	Prescribed intensity	Monitoring tools
Warm-up	30 s dynamic warm-up	50%–70% HRmax; RPE: 9–11	First Beat Bodyguard 2 & Huawei Watch 4 Pro; Borg’s RPE scale
Stair climbing (work bout)	30 s all-out stair climbing	80%–95% HRmax; RPE: 15–18	
Active recovery	60 s active recovery	50%–70% HRmax; RPE: 9–11	

HRmax, age-predicted maximal heart rate; RPE, Borg Rating of Perceived Exertion scale (6–20). Exercise intensity will be monitored using heart-rate data from a chest-worn electrocardiography (ECG)-based monitor (Firstbeat Bodyguard 2) and a wrist-worn photoplethysmography (PPG)-based smartwatch (Huawei Watch 4 Pro), supplemented by post-session RPE.

- The Firstbeat Bodyguard 2 device (Firstbeat Technologies Ltd., Jyväskylä, Finland) will be used as the primary heart-rate recording device, and the HUAWEI WATCH 4 Pro (Huawei Technologies Co., Ltd., Shenzhen, China) will be used for real-time heart-rate feedback during supervised sessions.
- During warm-up and recovery periods, participants will be instructed to maintain a light-to-moderate intensity corresponding approximately to 50%–70% of predicted HRmax. During each stair-climbing set, the target intensity will be 80%–95% of predicted HRmax (49).
- Before each session, participants will complete a standardized 30-s dynamic warm-up consisting of 10 s of jumping jacks, 10 s of high knees, and 10 s of tuck jumps.
- All sessions will be supervised by qualified exercise instructors. Immediately after each session, subjective exertion will be recorded using the Borg 6–20 Rating of Perceived Exertion (RPE) scale (50).

#### Criteria for discontinuing or modifying allocated interventions

The intervention will be promptly discontinued if a participant encounters any serious adverse event (SAE), including exercise-induced palpitations, chest pain, or syncope, upon verification by the study medical team. Additionally, participants retain the entitlement to exit the study at any point without providing any reason.

#### Strategies to improve adherence to interventions

Prior to intervention initiation, all personnel involved in implementation will receive standardized training and pilot testing from the study core team. Competency in intervention protocols, intensity control, and safety management will be verified through assessments while simultaneously evaluating program feasibility and safety. During the intervention period, adherence will be supported through standardized weekly WeChat reminders, instructor-recorded attendance, and prespecified make-up sessions where feasible. Make-up sessions will be arranged within the weekly training schedule and will not alter the prespecified post-intervention assessment window. The 5–6 person adherence groups will be used only for scheduling coordination and attendance reminders. These groups will not provide peer counselling, motivational coaching, competitive ranking, or additional social-support activities. All adherence-related contacts will follow standardized procedures and be recorded to minimize differential social-support effects (51). Post-intervention, strategies including health improvement reports, personalized training feedback, and monthly health advice dissemination will be implemented to facilitate long-term maintenance of health behaviors.

#### Relevant concomitant care permitted or prohibited during the trial

During the trial intervention period, participants will be asked to maintain their usual dietary patterns and routine curriculum-based activities. Dietary intake will be recorded using a standardized 3-day 24-hour dietary recall, including 2 weekdays and 1 weekend day (52). Total energy intake derived from the dietary recalls will be summarized by group and used to contextualize outcomes plausibly influenced by dietary intake. Compulsory physical education (PE) classes required by the university curriculum will be permitted and recorded, as these classes represent routine curriculum-based activities rather than additional voluntary structured exercise training. Participants will be asked not to initiate any additional structured exercise training outside compulsory PE classes and the allocated trial intervention during the study period. Recorded compulsory PE exposure will be summarized by group and considered when interpreting the trial findings.

#### Provisions for post-trial care

The interventions in this study pose a low risk of injury, and previous similar studies have rarely reported adverse events. To reinforce participant protection, all participants will be covered by accidental injury insurance to address potential intervention-related injuries during the trial. If someone becomes hurt, they will receive the medical help they need until all of their symptoms go away after the trial ends, and the costs will be covered by insurance. The research team will call participants three months after the trial to check their health and look for any long-term effects of the intervention.

### Outcomes

#### Primary outcome measure

The single primary outcome of this trial is CRF, operationalized as maximal oxygen uptake (VO_2_max).

##### Cardiorespiratory fitness

VO_2_max is the classical marker for evaluating CRF and an independent prognostic factor for cardiovascular disease (53, 54). VO2max will be assessed using a standardized graded exercise test (GXT) (55, 56). Before testing, the Cosmed K4b² Portable Metabolic System (COSMED S.R.L., Rome, Italy) and h/p/cosmos^®^ recommended system configuration (h/p/cosmos sports & medical gmbh, Germany) will be calibrated to ensure treadmill speed and incline accuracy within ±0.2 km/h and ±0.5%, respectively. Test termination criteria will include a respiratory exchange ratio (RER) >1.10, ≥95% of age-predicted maximal heart rate (57), a VO_2_ plateau (<2mL·kg^-^¹·min^-^¹ over 30s) (58), or participant request to stop. The mean VO_2_ over the final 30s will be recorded and interpreted according to ACSM guidelines (59).

#### Secondary outcome measures

Secondary outcomes will include mental health indicators, body anthropometry, body composition, blood pressure, and vital capacity. These outcomes will provide supportive evidence regarding the broader physical and psychological effects of the intervention.

##### Body anthropometry

Body mass index (BMI) is a classical marker of overall obesity and is positively associated with cardiovascular risk (60). Participants will fast for at least 4hours before measurement. Height and weight will be assessed with an ultrasonic height and weight scale (DHM-30, Zhengzhou Dingheng Electronic Technology Co., Ltd.), and BMI will be calculated as weight/height² (61). Waist circumference will be measured with a soft tape (Deli Group, Ningbo, China) at the midpoint between the lowest rib and iliac crest, with a precision of 0.1 cm, at the end of normal expiration. Hip circumference will be measured at the level of maximum gluteal protrusion (62). Both waist and hip circumferences will be measured three times, and the mean will be recorded.

##### Body composition

Body fat percentage is an independent predictor of metabolic syndrome and is positively associated with insulin resistance (63, 64). Body composition—including basal metabolic rate (BMR), body fat percentage (FAT%), fat-free mass (FFM), total body water (TBW), extracellular water (ECW), and intracellular water (ICW)—will be assessed using the Bodystat^©^ QuadScan 4000 (Bodystat Ltd, UK). Participants will rest for 15minutes before testing and avoid vigorous exercise, alcohol, food, and water intake for at least 2 hours. Measurements will be conducted supine with electrodes attached to standard sites, and participants will remain relaxed.

##### Blood pressure

Abnormal blood pressure in young adults damages endothelial function and serves as an early warning of cardiovascular risk (65). Participants will be advised to sleep adequately and avoid vigorous activity, caffeine, and alcohol before testing. Blood pressure will be measured using an electronic sphygmomanometer (Yuwell YE600F, Jiangsu Yuwell Medical Equipment Co., Ltd.). The cuff will be placed on the left upper arm at heart level. Three measurements will be taken at 1-minute intervals, with the mean systolic (SBP) and diastolic (DBP) pressures used for analysis.

##### Vital capacity

Reduced vital capacity compromises cardiopulmonary reserve and oxygen delivery, increasing cardiovascular disease risk; it is a key indicator linking respiratory and cardiovascular health (66, 67). Vital capacity will be assessed using an electronic spirometer (FCS-10000, Shanghai Yilian Medical Instrument Development Co., Ltd.). The mouthpiece will be disinfected before testing. Participants will stand upright, hold the handle, inhale maximally to total lung capacity, then exhale smoothly to residual volume. The test will be repeated twice, and the highest value (ml) will be recorded.

##### Mental health

Psychological outcomes will be assessed using validated Chinese-language instruments selected to capture prespecified psychological and psychosocial domains relevant to sedentary university students, including depressive symptoms, perceived stress, mood state, general well-being, sleep quality, perceived social support, resilience, and body appreciation. These outcomes will be interpreted according to a prespecified hierarchical framework rather than as a single composite construct, because the selected instruments assess distinct but related psychological and psychosocial dimensions.

Depressive symptoms and perceived stress will be treated as key secondary psychological outcomes and assessed using the Beck Depression Inventory-II (BDI-II) (68) and 10-item Perceived Stress Scale (PSS-10) (69), respectively. Mood state, general well-being, and sleep quality will be treated as supportive psychological outcomes and assessed using the Profile of Mood States (POMS) (70), General Well-Being Schedule (GWBS) (71), and Pittsburgh Sleep Quality Index (PSQI) (72). Perceived social support, resilience, and body appreciation will be treated as exploratory psychosocial indicators and assessed using the Multidimensional Scale of Perceived Social Support (MSPSS) (73), Resilience Scale (74), and Body Appreciation Scale-2 (BAS-2) (75). Baseline covariates will include demographic characteristics and health behaviors, including smoking, alcohol use, and sedentary time.

#### Exploratory outcome measures

Exploratory mechanistic outcomes will include glycolipid metabolism, inflammatory markers, and adipokines. These outcomes will be used to explore potential metabolic, inflammatory, and adipokine-related pathways through which stair-climbing ES may influence health.

All biological sample collection and analyses will be performed in line with standardized protocols. Specifically, a 10-hour fasting period for participants, venous blood sampling from the antecubital vein between 7:00 and 9:00 AM, and aliquoting into EDTA anticoagulant tubes or clot activator tubes based on assay specifications. To ensure sample quality, low-temperature centrifugation will be completed within 30 minutes post-collection. Serum and plasma will be separated, stored at -80 °C in ultra-low temperature freezers, and protected from repeated freeze-thaw cycles until batch analysis (76).

##### Glycolipid metabolism outcomes

Fasting blood glucose (FBG), fasting insulin (FINS), and HOMA-IR are early-warning indicators of prediabetes (77), and blood lipid levels are core risk factors for coronary heart disease (78). FBG will be measured using the hexokinase method (Hitachi 7180 Biochemistry Analyzer, Japan); FINS by electrochemiluminescence immunoassay (ECLIA; Roche Cobas e601); and HOMA-IR will be calculated as [FINS (μU/mL) × FBG (mmol/L)] / 22.5 (79). Lipids will include total cholesterol (TC; CHOD-PAP), triglycerides (TG; GPO-PAP), HDL-C (direct method), and LDL-C (homogeneous direct method). All assays will be calibrated according to IFCC standards.

##### Inflammatory markers

High-sensitivity C-reactive protein (hs-CRP >3mg/L) indicates elevated atherosclerotic risk (80), while persistently high levels of interleukin-6 (IL-6) and tumor necrosis factor-alpha (TNF-α) promote atherosclerosis progression and cardiovascular disease (81). Hs-CRP, IL-6, interleukin-10 (IL-10), and TNF-α will be measured using magnetic particle chemiluminescence (Beckman Coulter Access kits) on the DXI 800 analyzer. Detection ranges and limits: hs-CRP 0.1–10mg/L (0.03mg/L), IL-6 1.5–500pg/mL (0.7pg/mL), IL-10 1.2–1000pg/mL (0.6pg/mL), TNF-α 1.7–1000pg/mL (0.8pg/mL). Each batch will include calibrators and quality controls (R² ≥0.995).

##### Adipokines

Elevated leptin promotes visceral fat accumulation and insulin resistance (82), whereas low adiponectin is associated with exacerbated metabolic dysfunction (83). The leptin-to-adiponectin ratio (LAR) will be calculated as leptin divided by adiponectin and used as an exploratory adipokine-balance index (84). Leptin and adiponectin will be measured by CMIA (Abbott ARCHITECT i2000SR), and LAR calculated directly. Detection ranges: leptin 0.1–100ng/mL (0.05ng/mL), adiponectin 1–30μg/mL (0.5μg/mL). Samples will be recentrifuged 5minutes before testing to remove precipitates. Assays will follow manufacturer instructions with strict incubation control (30minutes at 37°C).

### Participant timeline

This study will include two main assessment time points: baseline and post-intervention. The primary outcome, secondary outcomes, exploratory outcomes, and descriptive data will be collected at these time points. Each assessment phase will be completed within one week to minimize temporal variation in outcome measurement. For participants in the ES group, post-intervention VO_2_max assessment will be scheduled 48–72 h after the final completed intervention session, including any make-up session, and this interval will be recorded.

### Sample size

The sample size was calculated using VO_2_max as the single primary outcome. VO_2_max was selected because CRF is the principal physiological endpoint of the stair-climbing ES intervention. Previous stair-climbing ES evidence has reported improvements in peak oxygen uptake (VO_2_peak) among sedentary young adults, supporting CRF as a relevant physiological endpoint for the present trial (36).

The numerical assumptions for the sample size calculation were primarily informed by a previous 12-week stair-climbing ES RCT in sedentary obese college students (42). That trial reported post-intervention VO_2_max values of 37.61 ± 3.87 and 32.06 ± 4.95 mL·kg^-^¹·min^-^¹ in the intervention and control groups, respectively (42).

Based on the pooled post-intervention SD of 4.44 mL·kg^-^¹·min^-^¹, we prespecified a target between-group of 3.5 mL·kg^-^¹·min^-^¹. This target difference was smaller than the observed between-group difference of 5.55 mL·kg^-^¹·min^-^¹ in the source trial and corresponds to approximately 1 metabolic equivalent (MET) (85). However, because the pooled SD was derived from a small RCT in sedentary obese college students, the resulting standardized effect size should be interpreted as a pragmatic planning estimate rather than a population-specific estimate for the broader sedentary university-student population. The assumed standardized effect size was Cohen’s d = 0.79.

Using G*Power version 3.1, with a two-sided alpha level of 0.05, 80% power, and a 1:1 allocation ratio, 26 participants per group were required. Allowing for 10% attrition increased the required sample size to 29 participants per group. The registered target sample size was set at 30 participants per group, yielding a total planned sample of 60 participants.

### Recruitment

This study will enlist qualified students from Shihezi University. A multi-channel recruitment strategy that uses both online and offline methods will be used. The university’s official website and WeChat public account will post online job openings. Offline job openings will be posted in public areas on campus, such as libraries, gyms, dorms, and classrooms. Everyone who wants to participate must sign up using the link or contact information given in the announcements and posters. After they sign up, they will go through the established eligibility criteria screening process.

### Assignment of interventions: allocation

#### Sequence generation

An independent researcher not involved in recruitment, baseline assessment, or intervention delivery will generate the allocation sequence using R software (version 4.4.1), with a 1:1 allocation ratio. The sequence will be generated before enrolment and retained in a password-protected file by the independent sequence custodian.

#### Concealment mechanism

Allocation will be concealed using sequentially numbered, opaque, sealed envelopes prepared by personnel independent of recruitment and assessment. Each envelope will contain the group assignment and participant identification number only. Envelopes will remain sealed until completion of baseline assessment for the corresponding participant.

#### Implementation

After baseline assessment, the next sequentially numbered envelope will be opened by an independent researcher not involved in recruitment or outcome assessment. The allocation date, participant identification number, and assigned group will be documented in an allocation log.

### Assignment of interventions: blinding

#### Who will be blinded

This trial will use blinded outcome assessment and blinded data analysis. Participants and exercise instructors cannot be blinded because the intervention involves supervised stair-climbing exercise. This limitation may introduce performance bias. To reduce detection and analytical bias, CRF test technicians, blood sample analysts, psychological evaluators, and data analysts will remain blinded to group allocation. Participants and instructors will be instructed not to disclose allocation status during assessments. Blinding-specific training will cover procedures for maintaining masking, including avoiding questions about group allocation or intervention participation, restricting access to allocation records, using assessment forms and analysis datasets without group labels, and documenting any accidental disclosure of allocation as a potential unblinding incident. Blinding will be maintained through data de-identification, procedural separation, standardized assessments, and blinding-specific training.

#### Procedure for unblinding if needed

Unblinding will be initiated only in the event of a serious adverse event requiring knowledge of allocation for participant safety. The specific procedure is as follows:

- The attending physician completes a written unblinding request justifying its necessity.
- Upon approval by the principal investigator and the chair of the data safety monitoring board, the random sequence custodian opens the envelope to obtain allocation information.
- The ethics committee must be informed within 24 hours of unblinding. The participant will move to open-label management, and a backup team will do the next evaluations. The data will be marked as “unblinded” and added to the set for the intention-to-treat analysis.

### Data collection and management

#### Plans for assessment and collection of outcomes

This study will use paper case report forms as source documents during on-site assessments. Manually recorded outcome measurements will undergo point-of-assessment verification: one recorder will enter the value on the case report form, and a second recorder will verify it against the instrument display, assessor reading, or source measurement record before the participant leaves the assessment station. Immediate discrepancies will be resolved against the source measurement and documented where appropriate.

After on-site assessments, data from the paper case report forms will undergo independent double data entry into a password-protected Microsoft Excel 365 workbook. Discrepancies between the two electronic entries will be resolved against the original case report forms and documented in a correction log. Because direct electronic data capture will not be used, transcription error risk will be minimized through point-of-assessment source-value verification, independent double data entry, source-data reconciliation, and correction logging.

#### Plans to promote participant retention and complete follow-up

To promote retention and completion of scheduled procedures, standardized weekly WeChat reminders will be sent to participants. Telephone contact will be used for non-response, missed assessments, or three consecutive missed intervention sessions. All retention contacts will be documented in a retention log. Assessment sessions will be scheduled flexibly where possible. Small non-coercive completion incentives, such as certificates of participation or study-related gifts, will be provided after completion of trial assessments.

#### Data management

This study will use a three-stage data quality-control workflow:

- First, point-of-assessment verification will be conducted using the dual-recorder procedure described above to reduce errors in source-data recording during on-site assessments.
- Second, completed case report forms will be reviewed after each assessment day for completeness, legibility, and format consistency. Missing, ambiguous, or inconsistent entries will be queried and resolved against the source documents before data entry where possible.
- Third, independently entered electronic datasets will be compared and reconciled against the original case report forms. A designated quality-control team will conduct weekly range, consistency, and logic checks according to prespecified validation rules. All corrections will be traceable and documented in the correction log.

Paper-based data will be stored in locked cabinets under the custody of designated personnel, whereas electronic data will be encrypted.

#### Confidentiality

Neither the paper-based nor the digital datasets will contain any individual identifiers. Participants will receive a unique research identifier to ensure data anonymity.

### Statistical methods

All statistical analyses will be conducted in R (version 4.4.1). Continuous variables will be summarised as mean ± standard deviation (SD) or median [(IQR)], as appropriate, and categorical variables as n (%). Baseline characteristics will be described descriptively for the randomised sample.

The primary analysis will follow the intention-to-treat principle. The between-group effect on post-intervention VO_2_max will be estimated using analysis of covariance (ANCOVA), with group allocation as the fixed factor and baseline VO_2_max as the covariate. The primary treatment effect will be reported as the adjusted mean difference with a 95% confidence interval (CI), and statistical inference for the primary outcome will be based on a two-sided alpha level of 0.05. If meaningful between-group imbalance in recorded compulsory PE exposure is observed, a sensitivity analysis additionally adjusted for PE exposure may be conducted to examine the robustness of the primary findings.

Secondary and exploratory outcomes will be analyzed using analogous baseline-adjusted models. Psychological outcomes will be interpreted according to the prespecified hierarchy, with BDI and PSS prioritized as key secondary psychological outcomes. Outcomes with marked skewness will be analyzed using log-ANCOVA, with exponentiated estimates reported as geometric mean ratios and percentage differences. These outcomes will be interpreted as supportive or hypothesis-generating, with emphasis on effect estimates and 95% CIs rather than confirmatory hypothesis testing. Multiplicity considerations for prespecified secondary outcome domains will be addressed in the analysis hierarchy described for those outcomes.

Missing outcome data will be handled using multiple imputation with predictive mean matching, and estimates will be pooled using Rubin’s rules. The imputation model will include group allocation, the corresponding baseline outcome value, and relevant baseline predictors. A completers-only analysis will be performed as a sensitivity analysis. All tests will be two-sided.

### Oversight and monitoring

#### Composition of the coordinating center and trial steering committee

The central coordination unit will include data collection staff and research personnel tasked with recruiting participants, collecting data, and implementing interventions. The trial oversight committee will consist of the lead investigator and collaborating investigators, with responsibilities including finalizing the intervention protocol, personnel training, and overall trial supervision.

#### Composition of the data monitoring committee, its role and reporting structure

Given the low-risk nature, short duration, and modest sample size of this trial, a formal independent Data Monitoring Committee was not considered necessary. Safety oversight will be undertaken by the principal investigator and the trial steering team, who will review participant safety, protocol adherence, and any adverse events throughout the study. Any serious adverse event will be managed according to the prespecified emergency response procedures and reported to the ethics committee as required.

#### Adverse event reporting and harms

**Figure 4:**
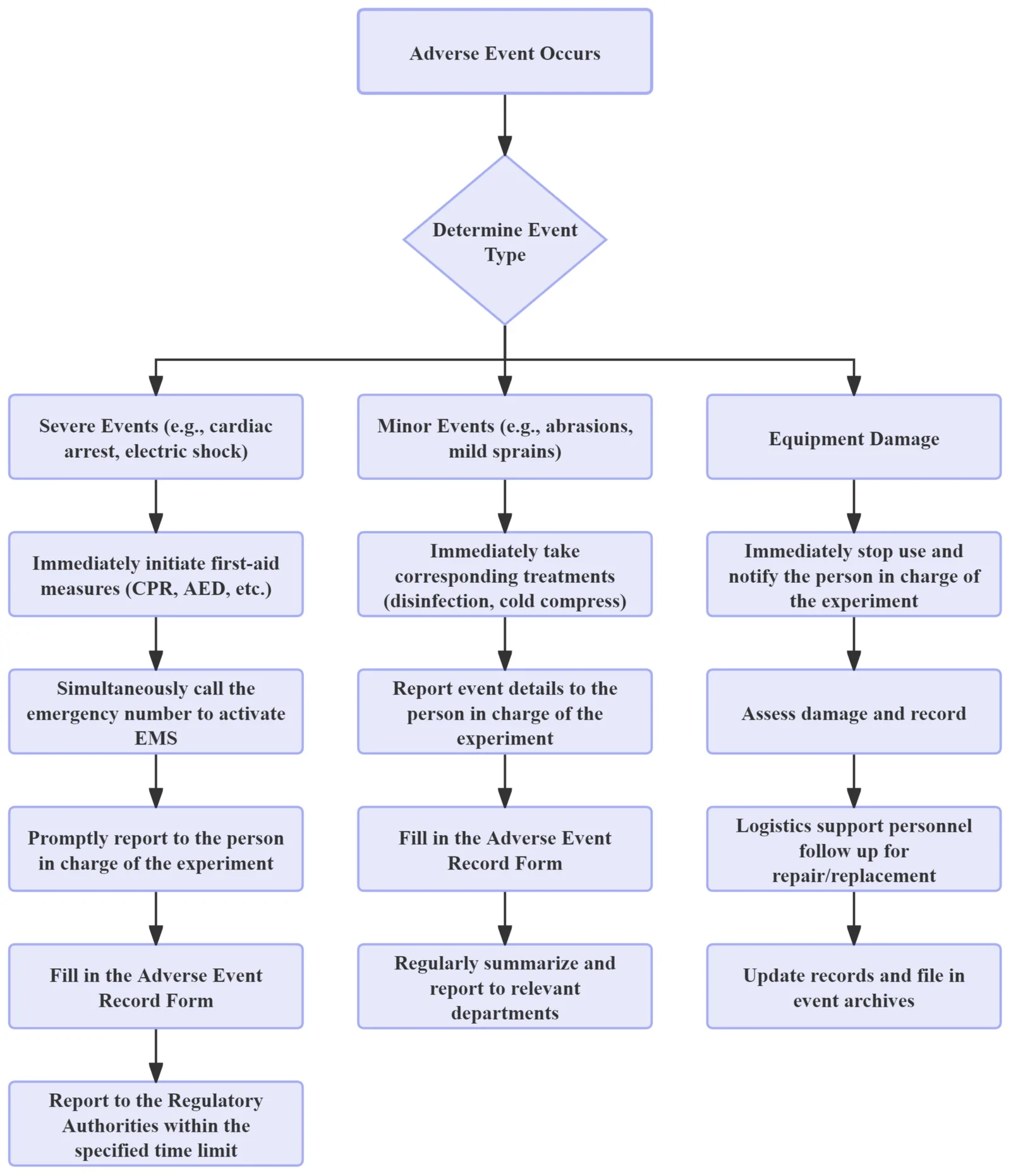
Adverse event management flowchart. The diagram summarizes procedures for classifying and managing severe events, minor events, and equipment damage during the trial. Severe events require first-aid measures, emergency medical activation, reporting, documentation, and regulatory reporting within the specified time limit. Minor events and equipment damage are managed through treatment or suspension, reporting, documentation, and record archiving. AED, automated external defibrillator; CPR, cardiopulmonary resuscitation; EMS, emergency medical services.

To prevent and address potential acute health risks during the intervention, an emergency response protocol and adverse event recording form have been established in addition to insurance coverage. This protocol seeks to guarantee participant safety and uphold study compliance by implementing standardized procedures for the collection, evaluation, management, and reporting of adverse events. **Figure 4** shows that information is gathered through on-site observation and participant reports, and then it is sorted by severity. Severe events require immediate first aid and activation of Emergency Medical Services (EMS), while non-severe events are managed on-site. All events must be documented using the recording form, with severe events reported to the ethics committee within a specified timeframe and non-severe events summarized periodically.

#### Frequency and plans for auditing trial conduct

The principal investigator will perform routine site inspections to monitor intervention progress. Subsequently, periodic reports will be submitted to the trial oversight committee and central coordination unit to evaluate intervention effectiveness.

#### Plans for communicating important protocol amendments to relevant parties

Any revisions of the study protocol must be filed by the lead investigator and approved by the Ethics Review Committee of Dongguan Seventh People’s Hospital before implementation.

## Discussion

Prolonged sedentary behavior in university students is associated with adverse health outcomes. Although ES and related short-bout accumulated exercise approaches have been proposed to improve health through physiological pathways such as metabolic regulation and cardiorespiratory stimulation, most evidence derives from studies in healthy adults and older populations, with limited data in university students. Existing ES trials have mainly examined short-term physiological effects while neglecting behavioral maintenance mechanisms (e.g., subjective experience, contextual feasibility), which are essential for sustaining long-term benefits. To address this gap, this protocol outlines a campus-based, structured stair-climbing ES intervention within the broader framework of short bouts of accumulated exercise designed to evaluate health outcomes, a multidimensional set of mental-health outcomes, and adherence-related responses among university students.

The distinctive contribution of this protocol lies in the population- and outcome-specific application of a structured stair-climbing ES intervention in sedentary university students, with concurrent assessment of CRF, selected physical-health indicators, multidimensional psychological outcomes, and adherence-related responses. The intervention will be conducted in designated stairwell spaces using prespecified short stair-climbing sessions consisting of 30-second vigorous stair-climbing sets separated by 60-second active recovery, with sessions completed under instructor supervision and within predefined campus time windows. Randomized allocation, allocation concealment, blinded outcome assessment, and blinded data analysis are incorporated as standard RCT procedures to support internal validity. This design is intended to improve protocol transparency and reproducibility and to inform the development of campus-based ES protocols for sedentary students. The intervention is therefore expected to increase brief vigorous activity exposure and partially interrupt sedentary time on intervention days, rather than throughout the week.

Several limitations warrant careful consideration. The sample size calculation was informed by ES-related VO_2_max evidence from a small RCT in sedentary obese college students. Greater VO_2_max variability in the broader sedentary university student population may reduce the actual statistical power. Eligibility screening based on self-reported IPAQ data may introduce assessment bias in physical activity and sedentary-time classification. The ≥6 h/day sedentary-time criterion was based on dose–response evidence from middle-aged and older adults and was applied to the present university-student sample by extrapolation. Anthropometric measures, including BMI and waist circumference, will be reported as baseline health characteristics rather than used to verify physical activity or sedentary behavior. Device-based verification of sedentary behavior and free-living physical activity is not planned in the present trial, which may increase the risk of sedentary-time misclassification and undetected activity contamination or co-intervention. The 12-week intervention period overlapping with final examination weeks may cause irregular training intervals or adherence fluctuations due to academic stress, although post-intervention VO_2_max assessment will be standardized according to the final completed intervention session. As a short-term study, the 12-week observation window might not fully capture the longer-term effects of ES, necessitating extended follow-up periods to assess effect persistence.

Despite these limitations, if supported, this trial would provide preliminary evidence on a structured stair-climbing ES protocol for sedentary university students and help clarify whether changes in CRF are accompanied by psychological and adherence-related responses. Subsequent studies should incorporate wearable sensors, including triaxial accelerometers, to improve sedentary-time classification and objectively quantify movement patterns. Such data may help examine whether intervention effects differ across BMI categories. Future studies should further examine personalized exercise dosing, dose-response relationships, comparative efficacy of diverse exercise snack formats, and combined interventions integrating ES with mindfulness-based practices.
